# Factors influencing community pharmacists’ knowledge about women’s issues in epilepsy

**DOI:** 10.3389/fpubh.2023.1251393

**Published:** 2023-09-08

**Authors:** Ammar Abdulrahman Jairoun, Sabba Saleh Al-himyari, Moyad Shahwan, Nageeb Hassan, Saleh AL-Tamimi, Maimona Jairoun, Saed H. Zyoud, Abdullah S. Alshehri, Mustfa Faisal Alkhanani, Reem Hasaballah Alhasani, Adnan S. Alharbi, Fahad S. Alshehri, Ahmed M. Ashour, Nasser M. Alorfi

**Affiliations:** ^1^Health and Safety Department, Dubai Municipality, Dubai, United Arab Emirates; ^2^Discipline of Clinical Pharmacy, School of Pharmaceutical Sciences, Universiti Sains Malaysia, Pulau Pinang, Gelugor, Malaysia; ^3^Pharmacy Department, Emirates Health Services, Dubai, United Arab Emirates; ^4^College of Pharmacy and Health Sciences, Ajman University, Ajman, United Arab Emirates; ^5^Centre of Medical and Bio-allied Health Sciences Research, Ajman University, Ajman, United Arab Emirates; ^6^Faculty of Pharmacy, Aden University, Aden, Yemen; ^7^Department of Clinical and Community Pharmacy, College of Medicine and Health Sciences, An-Najah National University, Nablus, Palestine; ^8^Clinical Research Centre, An-Najah National University Hospital, Nablus, Palestine; ^9^Pharmaceutical Care Services, King Abdulaziz Medical City, Ministry of National Guard – Health Affairs, Jeddah, Saudi Arabia; ^10^Department of Biology, College of Sciences, University of Hafr Al Batin, Hafr Al Batin, Saudi Arabia; ^11^Department of Biology, College of Applied Science, Umm Al-Qura University, Makkah, Saudi Arabia; ^12^Department of Clinical Pharmacy, College of Pharmacy, Umm Al-Qura University, Makkah, Saudi Arabia; ^13^Pharmacology and Toxicology Department, College of Pharmacy, Umm Al-Qura University, Makkah, Saudi Arabia

**Keywords:** women’s issues in epilepsy, community pharmacist, knowledge, antiepileptic drugs, epilepsy, optimal seizure control

## Abstract

**Background:**

Previous studies have highlighted instances where pharmacists lacked knowledge regarding women’s health issues related to epilepsy.

**Objectives:**

To assess UAE community pharmacists’ knowledge, toward women’s issues in epilepsy.

**Methods:**

a cross-sectional research method was employed. A team of seven pharmacy students in their final year visited a randomly selected sample of community pharmacies in the UAE and face-to-face interviews were conducted with the pharmacists using a structured questionnaire. The questionnaire includes two parts; Eight questions designed to elicit data about the demographics of the study participants and 12 questions eliciting insights into the participants’ knowledge of women’s issues in epilepsy.

**Results:**

A total of 412 community pharmacist were recruited in the study. The overall level of knowledge about women’s issues in epilepsy was good and the average knowledge score was 81% with a 95% confidence interval (CI) [79.1, 82.7%]. The results of multivariate analysis showed higher knowledge scores in chain pharmacies (OR 1.37; 95% CI 1.12–1.67), Chief pharmacists (OR 1.44; 95% CI 1.01–2.06), Pharmacists in charge (OR 3.46; 95% CI 2.7–4.45), pharmacists with 1–5 Years of experience (OR 2.87; 95% CI 1.71–4.82), pharmacists with 6–10 Years (OR 2.63; 95% CI 1.58–4.38), pharmacists with >10 years (OR 3.13; 95% CI 2.03–4.83), graduation form regional universities (OR 1.37; 95% CI 1.12–1.67), graduation form international universities (OR 1.73; 95% CI 1.36–2.20) and receiving a training on epilepsy (OR 1.36; 95% CI 1.12–1.67).

**Conclusion:**

While the findings reveal an overall promising level of knowledge among community pharmacists regarding the issues faced by women with epilepsy, pinpointing which clinical and demographic factors have the most significant impact on this knowledge would permit the implementation of tailored educational interventions. Workshops and modules targeting the issues faced by women with epilepsy would further raise the knowledge and competence among community pharmacists in this area, ensuring better pharmaceutical care for this population.

## Introduction

Epilepsy remains a prevalent neurological disorder in the United States and worldwide (1). Approximately 3.4 million Americans are living with active epilepsy (2). Globally, over 50 million individuals have this condition, with over 4.7 million living in the World Health Organization’s Eastern Mediterranean Region (3–5). This multifaceted disorder affecting the central nervous system is characterized by recurrent, unprovoked seizures not incited by an acute neurologic or systemic insult (6). Various interventions may be required to manage epilepsy, including antiepileptic drugs (AEDs), surgical procedures, and non-pharmacological approaches, such as vagus nerve stimulation. AEDs have emerged as the primary therapeutic strategy for most patients (7–10). Crucially, the use of AEDs carries inherent risks that must be acknowledged. Data analysis from the 2013 and 2015 Centers for Disease Control and Prevention (CDC) National Health Interview Surveys revealed that 90% of United States adults with self-reported active epilepsy were prescribed AEDs (11), with only 44% showing seizure control (6). Epilepsy accounted for $15.5 billion in annual direct and indirect costs, according to a 2010 CDC report (12).

Although epilepsy impacts genders equally (13), delivering health care services to women with epilepsy frequently encounters many specific challenges (14). These typically relate to choosing the AEDs for women of childbearing age because some have been showed to be teratogenic, interact with sex hormones (pre- and post-menopause), diminish contraceptive efficacy, induce sexual dysfunction in women, decrease bone mass, affect breastfeeding, and present various other concerns (14–16). Community pharmacists can recognize women diagnosed with epilepsy, implement interventions, and offer education addressing women’s concerns in epilepsy (17, 18). According to a recent review, the most frequently reported services performed by pharmacists for persons with epilepsy was medication management, with education and counseling ranking second and third. Only a few articles examined the use of care coordination documentation tools by physicians or pharmacists when caring for epilepsy patients. However, the review found that community pharmacies often go beyond dispensing medication when serving individuals with epilepsy. The above findings underline the lack of empirical research on the contributions made by community pharmacists to epilepsy care and point the way for further investigation (17). Other research has revealed that community pharmacists can make a valuable contribution to patient care in cases of epilepsy by providing relevant information and assisting in the optimization of their treatment. Community pharmacists are key members of healthcare teams, and are crucial as patient counselors who can ensure that the condition is appropriately and promptly managed, e.g., by utilizing rescue medication coupled with an seizure action plan (SAP) or acute seizure action plan (ASAP). In particular, community pharmacists are key to overcoming the significant communication gaps among patients, physicians, caregivers, and other healthcare professionals. (18).

Community pharmacists can substantially contribute to detecting medications associated with a decreased seizure threshold. Upon identifying such agents, one intervention might involve liaising with the prescriber to assess the feasibility of transitioning the patient to a different medication (19, 20). For instance, a patient using tramadol for chronic pain management might be switched to another opioid that does not compromise the seizure threshold. Community pharmacists should also disseminate information on various pharmacological alternatives for epilepsy treatment, offer recommendations, and address drug interactions. Many AEDs either induce or auto-induce the metabolism of other medications, and some inhibit the metabolism of other drugs (11).

Through conversations with patients or their family members, community pharmacists can evaluate the appropriateness of an AED to ensure optimal seizure control. A discussion topic could involve determining whether the patient has been prescribed the proper seizure rescue medication. First-line agents for status epilepticus include benzodiazepines such as midazolam, diazepam, and lorazepam (21). Selecting the most suitable agent must consider the distinct pharmacokinetics of these benzodiazepines, as well as the administration route, to accommodate individual patient needs. Proactive intervention by community pharmacists to provide information about the importance of seizure medication treatment adherence could likely decrease seizure incidence. Approximately 70% of epileptic patients treated with AEDs could achieve seizure freedom by adhering to their most effective regimen (22). Community pharmacists are well-positioned to counsel patients on the potential adverse effects of some Food and Drug Administration–approved AEDs (23, 24).

More than half of surveyed women showed that the information from their health care providers on women’s issues in epilepsy was insufficient, and they expressed a desire for more comprehensive information from their attending physicians (14, 25). Multiple knowledge gaps were identified, including urgent situations causing immediate contact with the patient’s clinician (26). Patients reported that, despite maintaining a good relationship with their pharmacist, they had concerns about their lack of privacy and did not want to pay for additional services. Such feedback could pinpoint areas where pharmacists could assume a more extensive role in epilepsy management (27).

People with epilepsy in the UAE are generally prescribed antiepileptic drugs (AEDs) by neurologists or other healthcare providers who have a specialty in epilepsy management, accessible through public or private healthcare channels. For UAE nationals, AEDs are subsidized at public hospitals and clinics or are even offered free of charge, meaning patients can affordably access this vital medication. The UAE also runs several health insurance programs that reimburse residents for certain medical costs, and individuals and families can access these or choose to have private health insurance. The insurance policy and its provider will determine the level of coverage, but most health insurance plans at least partially reimburse the costs for prescribed medications, such as AEDs. To receive a reimbursement of eligible expenses, the patient must first pay for the medication and subsequently file a claim with their insurance provider.

Managing drug therapies for patients with epilepsy and prescribing AEDs in the UAE is a task for both community pharmacies and hospital pharmacies. Hospital pharmacies in particular have AEDs on standby to give inpatients who need immediate medical attention, as well as outpatients as part of follow-up care. Integrating hospital pharmacies and community pharmacies has produced a healthcare system that can comprehensively serve the needs of persons with epilepsy, from acute care to providing medication to ensuring the long-term management of the condition.

To ensure that the dispensed AEDs are safe and of a high quality, both community and hospital pharmacies are subject to strict government regulations and must meet licensing requirements. Licensed pharmacists are a critical part of medication management as they accurately dispense medication and ensure that patients have the necessary information on how to use the medication and how to follow the prescribed treatment regimens.

Based on the observed gap in the literature on this particular topic in the UAE, we contend that certain community pharmacists in the UAE, specifically those with more professional experience, graduates from regional or international universities, and those who have been trained in epilepsy management, display more knowledge of the issues that women with epilepsy face. By evaluating their knowledge and identifying the key factors affecting their understanding of this topic, this work aims to fill the substantial gaps in the knowledge and lead to the betterment of pharmaceutical care for women with epilepsy. To our knowledge, this is the first study to assess UAE’s community pharmacists’ knowledge, to address the knowledge Gap and Factors Influencing Community Pharmacists’ toward women’s issues in epilepsy.

## Methods and materials

### Study setting and design

To assess community pharmacists’ awareness of women’s epilepsy-related issues, a cross-sectional research method was employed. From September 2022 to March 2023, a team of seven pharmacy students in their final year visited a randomly selected sample of community pharmacies in the UAE. They conducted face-to-face interviews with the pharmacists. To ensure accurate data collection, the students underwent comprehensive training on both the questionnaire and the study’s scientific jargon before commencing the interviews. Extensive training was deemed essential based on previous research, which highlighted its positive impact on interviewer competence and the reduction of survey errors. By following this rigorous approach, we aimed to get reliable and representative insights into community pharmacists’ awareness of women’s epilepsy-related issues.

### Research instrument development

A structured questionnaire was developed based on the insights generated from a literature review (14, 26, 27). To adapt the study to the UAE context and ensure its relevance, input from neurology experts was sought to review the questionnaire’s design and suitability. Six faculty members from Ajman University’s medicine and clinical pharmacy department were consulted to assess the questionnaire’s appropriateness and relevance. Their valuable feedback was incorporated into the questionnaire after minor revisions were made.

Based on the experts’ suggestions, several modifications were implemented in the questionnaire. These included providing clear definitions for scientific terminology, changing the numbering of questions and pages, replacing the field name “Sex” with “Gender” consistently, establishing connections between certain questions, and determining a specific termination point for the questionnaire.

To evaluate the questionnaire’s content validity, a Lawshe analysis was conducted prior to conducting the pilot test (28). During the content validity assessment, items with a content validity ratio (CVR) exceeding 0.78 were deemed acceptable, whereas items falling below this threshold were eliminated from the questionnaire. All items in the questionnaire surpassed the CVR benchmark of 0.78, showing satisfactory validity. To further evaluate the questionnaire’s content validity, the means of the items that met the acceptable CVR criteria were used in calculating the content validity index (CVI) for the final instrument. The resulting CVI value of 0.891 showed that the final questionnaire possessed satisfactory overall validity (29).

To assess the face validity of the instrument, pilot testing was conducted with a group of 25 community pharmacists between September 11 and September 20, 2022. The data collected from these participants was excluded from the final analysis. Out of the 25 participants, 23 successfully completed the survey, indicating a high completion rate. Following the pilot study, the size of the primary research sample was determined, considering the findings and insights gained from the pilot testing phase. Additionally, the questionnaire’s reliability was evaluated using Cronbach’s alpha, a measure of internal consistency. The resulting Cronbach’s alpha coefficient of α 0.79 indicated acceptable internal reliability.

### Research instrument sections

The study questionnaire comprised two parts:

- Eight questions designed to elicit data about the demographics of the study participants: gender, professional position (i.e., pharmacist in charge or chief pharmacist), years of professional experience, working hours/day, numbers of patients treated per day, and whether they had received training on epilepsy and antiepileptic drugs.
- Twelve questions eliciting insights into the participants’ knowledge of women’s issues in epilepsy.

### Questionnaire scoring

The questionnaire contained 12 items that assessed the participants’ knowledge about women’s issues in epilepsy. The responses were provided in a “Yes” or “No” format. Questions on knowledge had only one “correct” option; the rest were “incorrect” answers. One point was allocated for each correct answer and the knowledge score was determined by adding up the points for each question. The participants’ knowledge score was calculated by computing the median score to divide the knowledge scores into good knowledge.

- The median knowledge score was 10. As such, respondents who achieved a score of 10 or above were classified as having good knowledge, while those with a score below 10 did not.

### Sample size calculation

A pilot study was performed to determine the required sample size for the main research study. The overall response rate was 92%. Respondents were specifically asked, “Do you possess good skills and experience in counseling and health promotion for women’s issues in epilepsy?” Approximately half (50%) of the participants responded positively to this question. To ensure a high level of confidence, this research utilized a 5% alpha level, resulting in a 95% confidence interval (CI). The precision (D) was set at 5%, enabling a maximum CI width of 10%. Considering the assumed non-response rate of 20%, a sample size of 480 respondents was adequate.

### Target population

Several key factors influenced the selection of the primary research sample. Eligible participants for this study included community pharmacists who possessed a minimum of 3 months’ work experience in either independent or chain pharmacies registered with reputable healthcare authorities such as the Ministry of Health, the Health Authority Abu Dhabi (HAAD), or the Dubai Health Authority. Respondents who did not meet the specified criteria were excluded from the study. Specifically, individuals with less than 3 months of professional experience, those still on probation, individuals not registered with any of the mentioned health authorities, or those who had recently gained their qualification were disqualified from participation.

### Sampling technique

A stratified random sampling approach was employed to ensure the results of the study represented the population of interest. According to a 2010 survey, 2000 community pharmacists were active in the UAE (30). To ensure a comprehensive sample, we used multiple sources to get contact information for community pharmacies in the chosen regions of the UAE. We collected data from reliable sources, such as the Yellow Pages and local business directories. By organizing the community pharmacies into strata or groups based on their locations, we implemented a stratified sampling approach. This resulted in three distinct strata: community pharmacies in Dubai, community pharmacies in Abu Dhabi, and community pharmacies in the Northern Emirates.

Once the community pharmacies were selected, we meticulously recorded all relevant information in an Excel spreadsheet, including the pharmacy’s name, location, type, phone number, and email address. Each pharmacy was assigned a unique ID number to maintain accuracy and facilitate identification. Subsequently, we randomly selected 480 community pharmacies using a straightforward random sampling method from the compiled list. The community pharmacies were chosen based on their type and location for organizational purposes.

### Data collection

Between 18th September 2022 and 28th March 2023, our team of trained researchers visited the selected community pharmacies in the UAE. The purpose of the research was explained to the pharmacists at each pharmacy, and their cooperation was sought. During these visits, the researchers requested the pharmacists’ email addresses to facilitate further communication. Following the initial introduction and data collection, face-to-face interviews were conducted with the pharmacists using a structured questionnaire.

### Statistical analysis

The data collected was analyzed using SPSS Version 26, a widely used statistical software package. The continuous quantitative variables, which followed a normal distribution, were described using the mean and standard deviation (SD). Categorical variables were summarized using frequencies and percentages. To identify potential differences between the groups, various statistical tests were employed based on the variables. Unpaired student t-tests, one-way ANOVA, and non-parametric tests were used to compare quantitative variables. Prior to conducting these tests, the normality assumption was assessed using the Shapiro–Wilk test (with a value of p greater than 0.05 showing normal distribution) or by visually inspecting a Normal Q-Q Plot. In order to determine the factors influencing community pharmacists’ expertise, multivariate logistic regression models were employed. This allowed for the identification of significant predictors while controlling for other variables. Statistical significance was considered for *p*-values below 0.05, showing a reliable association or difference between variables.

### Ethical considerations

This study received ethical approval from the Institutional Ethical Review Committee at Ajman University, under the reference number P-H-S-2022-2-14. Prior to beginning data collection, all participants were provided with clear information about the study’s objectives. They were informed that their voluntary and complete consent was essential for participating and completing the questionnaire. Written informed consent was got from all respondents, ensuring their understanding and agreement to participate.

To protect the privacy and confidentiality of the participants, their identities were not recorded. Stringent measures were implemented to ensure the anonymity of the respondents. Confidentiality was maintained throughout the study, and data were securely stored and accessible only to the research team.

## Results

### Demographic information of the study sample

A total of 412 community pharmacist were recruited in the study. About half participants (51.9%) were male and 56.8% aged 25–34 years. Chain pharmacies constituted 68.2% of the study sample and 31.8% were independent pharmacies. The position of the pharmacist was: 50% (n = 206) were assistant pharmacist, 10.2% (*n* = 42) were Chief pharmacists and 39.8% (n = 164) were pharmacists in charge. Of the total, 206 (50%) had <1 year experience, 27 (6.6%) 1–5 years of experience, 34 (8.3%) 6–10 years of experience and 145 (35.2%) > 10 years of experience. Among the total participants, 23.8% graduated from local universities, 26.2% graduated from regional universities and 50% graduated from international universities (Table 1).

**Table 1:** Number and percentages of the questions on demographic information (*n* = 412).

Demographics	Groups	Frequency	Percentages
Gender	Male	214	51.9%
	Female	198	48.1%
Age group	25–34	234	56.8%
	35–44	132	32%
	≥ 45	46	11.2%
Pharmacy type	Independent pharmacy	131	31.8%
	Chain pharmacy	281	68.2%
Position in the pharmacy	Assistant pharmacist	206	50%
	Chief pharmacist	42	10.2%
	Pharmacist in charge	164	39.8%
Work experience	< 1 year	206	50%
	1–5 years	27	6.6%
	6–10 years	34	8.3%
	> 10 years	145	35.2%
Graduation university	Local	98	23.8%
	Regional	108	26.2%
	International	206	50%
Trained on epilepsy and antiepileptic drugs study year recoding	No	131	31.8%
	Yes	281	68.2%

DM, diabetes mellitus.

### Assessment of knowledge about women’s issues in epilepsy among community pharmacists

The overall level of knowledge about women’s issues in epilepsy was good. The average knowledge score was 81% with a 95% confidence interval (CI) [79.1, 82.7%]. The results pf each question related to knowledge about women’s issues in epilepsy were shown in Table 2.

**Table 2:** Number and percentages of the questions on knowledge about women’s issues in epilepsy.

Knowledge items	Correct response	Incorrect answer		Correct Answer	
		*F*	%	*F*	%
1-In terms of the menstrual cycle, endogenous estrogen is known to have proconvulsant properties, while progesterone has anticonvulsant properties	Yes	60	14.6	352	85.4
2-Women with epilepsy are more likely to experience sexual dysfunction than those without epilepsy	Yes	79	19.2	333	80.8
3-Certain antiepileptic drugs are linked to osteomalacia (reduced bone mass)	Yes	55	13.3	357	86.7
4-Switching to a different form of the same antiepileptic drug can trigger increased side effects or seizures	Yes	82	19.9	330	80.1
5-A 200-mg dose of topiramate triggers enzymatic induction, and thus may diminish the effectiveness of certain contraceptives	Yes	145	35.2	267	64.8
6- The effectiveness of certain contraceptives can be reduced by antiepileptic drugs that induce enzymes	Yes	101	24.5	311	75.5
7-Women who have epilepsy and who become pregnant should discontinue their antiepileptic medication	No	35	8.5	377	91.5
8- The best antiepileptic medication for a woman who is pregnant is that which is most suitable for her syndrome and type of seizure	Yes	81	19.7	331	80.3
9- In most cases, the children of women with epilepsy are healthy	Yes	124	30.1	288	69.9
10- Exposure to valproic acid during pregnancy does not increase the risk of the child developing autism	No	114	27.7	298	72.3
11- Folic acid consumed by women on antiepileptic medication before and during pregnancy can reduce the risk of teratogenesis in children	Yes	32	7.8	380	92.2
12- For most women who are on antiepileptic medication, it is safe to breastfeed	Yes	35	8.5	377	91.5

F, frequency; %, percentages.

There was a statistically significant relationship between knowledge about women’s issues in epilepsy and the following factors: gender (*p* = 0.005), age group (*p* = 0.001), pharmacy type (*p* = 0.014), position in the pharmacy (*p* = 0.003), work experience (*p* < 0.001), Graduation university (*p* < 0.001) and receiving a training on epilepsy and antiepileptic drugs (*p* < 0.001) (Table 3).

**Table 3:** Community pharmacists’ knowledge according to demographics.

Demographics		Knowledge scores (12 items)			
	*N*	Mean	95% CI		*P*-value
Gender					
Male	214	9.41	9.09	9.73	0.005*
Female	198	10.04	9.74	10.33	
Age group					
25–34	234	9.01	8.70	9.32	0.001*
35–44	132	10.53	10.20	10.84	
≥ 45	46	10.96	10.60	11.31	
Pharmacy type					
Independent pharmacy	131	8.32	7.91	8.73	0.014*
Chain Pharmacy	281	10.36	10.14	10.58	
Position in the pharmacy					
Assistant pharmacist	206	8.70	8.38	9.03	
Chief pharmacist	42	10.40	9.86	10.95	0.003*
Pharmacist in charge	164	10.79	10.55	11.05	
Work experiences					
< 1 year	206	8.70	8.38	9.03	<0.001*
1–5 Years	27	10.63	9.99	11.27	
6–10 Years	34	10.59	9.78	11.39	
> 10 years	145	10.76	10.52	11.01	
Graduation university					
Local	98	8.02	7.53	8.51	<0.001*
Regional	108	9.32	8.93	9.72	
International	206	10.72	10.49	10.94	
Trained on epilepsy and antiepileptic drugs					
No	131	8.32	7.91	8.73	<0.001*
Yes	281	10.36	10.14	10.58	

^*^P-values < 0.05 considered statistically significant, *P*-values obtained from independent *t* test and one way ANOVA.

### Factors associated with community pharmacists’ knowledge about women’s issues in epilepsy

The results of multivariate analysis showed higher knowledge scores in chain pharmacies (OR 1.37; 95% CI 1.12–1.67), Chief pharmacists (OR 1.44; 95% CI 1.01–2.06), Pharmacists in charge (OR 3.46; 95% CI 2.7–4.45), pharmacists with 1–5 Years of experience (OR 2.87; 95% CI 1.71–4.82), pharmacists with 6–10 Years (OR 2.63; 95% CI 1.58–4.38), pharmacists with >10 years (OR 3.13; 95% CI 2.03–4.83), graduation form regional universities (OR 1.37; 95% CI 1.12–1.67), graduation form international universities (OR 1.73; 95% CI 1.36–2.20) and receiving a training on epilepsy (OR 1.36; 95% CI 1.12–1.67) (Table 4).

**Table 4:** Regression analysis for the factors affecting the knowledge about women’s issues in epilepsy.

	Good knowledge ≥ 10			
Demographics	OR	95% CI		*P*-value
Gender (Ref. Male)				
Female	1.690	0.731	1.083	0.244
Age group (Ref. 25–34)				
35–44	1.212	0.575	1.146	0.508
≥ 45	1.162	0.744	1.815	0.235
Pharmacy type (Ref. Independent pharmacy)				
Chain Pharmacy	1.372	1.123	1.675	0.002*
Position in the Pharmacy (Ref. Assistant pharmacist)				
Chief pharmacist	1.446	1.011	2.068	0.043
Pharmacist in charge	3.469	2.703	4.453	0.001*
Experiences (Ref. < 1 year)				
1–5 Years	2.877	1.716	4.824	<0.001*
6–10 Years	2.637	1.585	4.387	<0.001*
> 10 years	3.136	2.032	4.838	<0.001*
Numbers of patients served daily (Ref. Local)				
Regional	1.372	1.123	1.675	0.002*
International	1.737	1.367	2.207	0.001*
Trained on the epilepsy (Ref. No)				
Yes	1.369	1.119	1.674	0.002*

*p-values less than 0.05 were considered statistically significant.

## Discussion

This study aimed to assess the level of knowledge among community pharmacists regarding women’s issues in epilepsy, specifically in the UAE.

Community pharmacists play a crucial role in supporting patients with chronic diseases, including women with epilepsy, because of their accessibility and proper training (31).

The results revealed that most participating pharmacists exhibited a good level of knowledge concerning women’s issues in epilepsy. However, previous studies conducted in different locations have highlighted instances where pharmacists lacked knowledge regarding women’s health issues related to epilepsy (26, 27).

Current study identified potential knowledge gaps among pharmacists regarding the interaction between enzyme-inducing antiepileptic drugs (AEDs) and the effectiveness of contraceptives.

This work reveals worrisome gaps in community pharmacists’ knowledge of two crucial issues facing women with epilepsy. To being with, only 64.8% of the surveyed community pharmacists had knowledge of the enzymatic induction sparked by a 200 mg dose of topiramate, which can cause a reduction in the effectiveness of some contraceptives; this suggests that the respondents are unaware of the drug interactions at play in this condition. This gap should raise alarm bells due to the severe implications for women with epilepsy who use hormonal contraceptives. To raise the knowledge level of pharmacists and enable them to accurately advise women taking topiramate to manage seizures, there need to be targeted education programs to train pharmacists on the updated guidelines regarding drug interactions.

The other important finding of this study is that only 69.9% of the surveyed community pharmacists understood that women with epilepsy generally have healthy children, highlighting a gap in the common understanding of the interactions between epilepsy and pregnancy. There is an urgent need to address the prevailing misconceptions on epilepsy and pregnancy as these are causing fear and anxiety among women with epilepsy who wish to bear children. Healthcare professionals, including pharmacists, can engage in educational interventions and provide more patient counseling to ensure that women with epilepsy have accurate information on managing their condition while pregnant. Filling these knowledge gaps will enable healthcare providers to act collectively and provide better pharmaceutical care for women with epilepsy, leading to improved health outcomes for this population overall.

It is crucial for pharmacists to be aware of these AED-contraceptive interactions, as a lack of such knowledge could lead to unintended pregnancies in women with epilepsy (32–35). In this study, pharmacists could identify the most important and common women’s issues in epilepsy. The average knowledge score obtained in this study was 81%, which was higher compared to other findings, where the median correct knowledge score was only 53.8% (36). Previous studies have also reported a fair level of knowledge regarding women’s general health issues among pharmacists (27, 36, 37).

One concerning finding from this study was that approximately 35% of pharmacists could not answer questions related to the dose of topiramate that triggers enzymatic induction. This result aligns with a previous study that surveyed pharmacists in Ohio (28) and physicians (37), where similar gaps in knowledge were observed, such as the impact of enzyme-inducing AEDs on contraceptive effectiveness and the potential consequences of generic substitution on seizure control or side effects.

Only 27.7% of pharmacists in this study were unaware that exposure to valproic acid during pregnancy does not increase the risk of the child developing autism. This finding is consistent with previous studies that have reported a lack of knowledge among pharmacists regarding the association between valproic acid and the risk of autism in offspring. Unfortunately, various studies, including those conducted in the region, have shown that individuals with epilepsy face stigma and negative attitudes from both society and some healthcare professionals (38–40). Pharmacists can play a crucial role in educating patients, their families, and society about epilepsy and its associated health issues.

Interestingly, this study found that pharmacists with more years of experience had better knowledge regarding women’s issues in epilepsy. This run simirarly with a recent study that found that respondents with at least 15 years of experience had significantly better knowledge (41). This result contrasts with several previous studies that showed no significant association between years of practice or higher education and pharmacists’ knowledge on women’s issues in epilepsy (27, 42). This optimistic finding suggests that experience may contribute to a better understanding of these specific issues.

The findings of this study show that receiving training specifically on epilepsy significantly improved pharmacists’ knowledge of women’s issues in epilepsy. It is important to acknowledge that challenges exist not only for women with epilepsy but also for healthcare professionals involved in their care (27, 43). Optimal therapy with appropriately selected antiepileptic drugs (AEDs) has the potential to achieve seizure freedom in 70–80% of adults with new-onset epilepsy (44, 45). Factors such as improper AED selection, non-adherence to AEDs, drug–drug interactions, and drug resistance can impact seizure control and the patient’s quality of life (46).

In current study setting, there is a need for targeted interventions to increase awareness among pharmacists regarding women’s health issues in pregnancy (27). Short courses on epilepsy management conducted by experts in the field have been suggested as a helpful approach to enhance pharmacists’ awareness of women’s health-related issues in epilepsy (27, 47). Increasing exposure to and interaction with individuals living with epilepsy may also contribute to enhancing pharmacists’ knowledge of specific issues related to women’s health in epilepsy. Implementing pedagogic and mentoring interventions specifically tailored to these topics could be beneficial. However, further investigations are necessary to assess the effectiveness of these interventions in terms of pharmacists’ knowledge and patient outcomes.

We acknowledge several limitations of current study. First, as this was a cross-sectional study, the regression models used cannot establish causality between the variables. Therefore, generalizing these findings over time may not be possible, and further longitudinal research is warranted in this area. Second, the survey responses relied on self-reporting, which may introduce biases such as social desirability and recall biases. Despite these limitations, the findings are robust considering the significant number of participating community pharmacists and the high response rate observed.

## Conclusion

While the findings reveal an overall promising level of knowledge among community pharmacists regarding the issues faced by women with epilepsy, pinpointing which clinical and demographic factors have the most significant impact on this knowledge would permit the implementation of tailored educational interventions. For instance, workshops and modules targeting the issues faced by women with epilepsy would further raise the knowledge and competence among community pharmacists in this area, ensuring better pharmaceutical care for this population. Community pharmacists are key to the provision of patient-centered care, and thus strengthening their understanding in this regard can have a significant positive influence on the quality of life for women with epilepsy in the UAE. In addition, there is a need to promote collaborative action between the healthcare sector, regulatory bodies and academia so that community pharmacists can have the best possible tools to meet the changing healthcare needs of this population.

## Data availability statement

The original contributions presented in the study are included in the article/supplementary material, further inquiries can be directed to the corresponding authors.

## Ethics statement

The studies involving humans were approved by this study received ethical approval from the Institutional Ethical Review Committee at Ajman University, under the reference number P-H-S-2022-2-14. Prior to beginning data collection, all participants were provided with clear information about the study’s objectives. They were informed that their voluntary and complete consent was essential for participating and completing the questionnaire. Written informed consent was got from all respondents, ensuring their understanding and agreement to participate. To protect the privacy and confidentiality of the participants, their identities were not recorded. Stringent measures were implemented to ensure the anonymity of the respondents. Confidentiality was maintained throughout the study, and data were securely stored and accessible only to the research team. The studies were conducted in accordance with the local legislation and institutional requirements. The participants provided their written informed consent to participate in this study.

## Author contributions

AJ, SA-h, and MS conceptualized the project, contributed to the data analysis, and interpretation. AJ, NH, FA, and NA contributed to the methodology development. AJ, MJ, and SA-T contributed to the data collection. MA, RA, SZ, and SA-T investigated and wrote the discussion. All authors developed, wrote, agreed, read, and approved the final manuscript.

## Conflict of interest

The authors declare that the research was conducted in the absence of any commercial or financial relationships that could be construed as a potential conflict of interest.

## Publisher’s note

All claims expressed in this article are solely those of the authors and do not necessarily represent those of their affiliated organizations, or those of the publisher, the editors and the reviewers. Any product that may be evaluated in this article, or claim that may be made by its manufacturer, is not guaranteed or endorsed by the publisher.
